# Competition between Intra and Intermolecular Triel Bonds. Complexes between Naphthalene Derivatives and Neutral or Anionic Lewis Bases

**DOI:** 10.3390/molecules25030635

**Published:** 2020-02-01

**Authors:** Wiktor Zierkiewicz, Mariusz Michalczyk, Steve Scheiner

**Affiliations:** 1 Faculty of Chemistry, Wrocław University of Science and Technology, Wybrzeże Wyspiańskiego 27, 50-370 Wrocław, Poland; mariusz.michalczyk@pwr.edu.pl; 2 Department of Chemistry and Biochemistry, Utah State University, Logan, UT 84322-0300, USA

**Keywords:** triel bond, intramolecular triel bond, MP2, MEP, π-hole

## Abstract

A TrF_2_ group (Tr = B, Al, Ga, In, Tl) is placed on one of the α positions of naphthalene, and its ability to engage in a triel bond (TrB) with a weak (NCH) and strong (NC^−^) nucleophile is assessed by ab initio calculations. As a competitor, an NH_2_ group is placed on the neighboring C^α^, from which point it forms an intramolecular TrB with the TrF_2_ group. The latter internal TrB reduces the intensity of the π-hole on the Tr atom, decreasing its ability to engage in a second external TrB. The intermolecular TrB is weakened by a factor of about two for the smaller Tr atoms but is less severe for the larger Tl. The external TrB can be quite strong nonetheless; it varies from a minimum of 8 kcal/mol for the weak NCH base, up to as much as 70 kcal/mol for CN^−^. Likewise, the appearance of an external TrB to a strong base like CN^−^ lessens the ability of the Tr to engage in an internal TrB, to the point where such an intramolecular TrB becomes questionable.

## 1. Introduction

Lewis acid–Lewis base interactions continue to be a heavily explored area of modern chemistry. The wide diversity of Lewis acids and bases involves them in numerous different chemical and biological processes [1,2,3,4,5,6,7,8,9,10,11,12]. Within this general topic, an enormous amount of research currently centers on the topic of σ-hole/π-hole interactions [13,14,15,16,17,18,19,20,21,22,23,24]. One aspect which makes these noncovalent interactions both surprising and unique is the attractive force between electronegative atoms, which simple chemical intuition would have guessed would be repulsive. The resolution of this paradox, initially explained for halogen atom–nucleophile contacts [25,26,27,28] is the anisotropy of the electron density around the halogen X atom. This density is thinned in the vicinity of the outer lobe of the *p* orbital engaged in the R-X covalent bond, which is commonly denoted as a σ-hole. There are also planar molecules in which an electron deficiency occurs above the plane, referred to as a π-hole [29,30,31,32,33,34,35,36,37,38,39,40,41,42]. Following the initial introduction of the σ-hole concept to rationalize the halogen bond, [43,44] the same idea has been expanded to various other groups of the periodic table [45], now denoted as chalcogen, pnicogen, tetrel, triel, and even aerogen bonds [46,47,48,49,50,51,52,53,54,55,56].

As triel (Tr) atoms (B, Al, etc) typically find themselves in planar TrR_3_ molecular arrangements, a π-hole can be found above and below this atom, which facilitates the formation of a triel bond (TrB) with an approaching nucleophile. There are also exceptional cases wherein a tetravalent Tr atom can generate a σ-hole [57]. This TrB has generated sufficient interest so as to be the focus of a number of prior quantum chemical studies [58,59,60,61,62,63,64,65,66,67,68,69,70,71,72,73]. Our own earlier study of the TrB [73] in complexes of TrR_3_ (Tr = B, Al, Ga; R = H, F, Cl, Br, CH_3_) with pyrazine provides some information about the influence of various substituents on the energy, geometry, and properties of this interaction. Complexation occurred in one of two ways, either through a Tr π-hole to the N-lone pair of pyrazine or via a stacked arrangement. The former was many times stronger than the latter. The two geometries also differed significantly in their fundamental nature: the stronger π-hole complex relied primarily on Coulombic forces and orbital interactions, whereas dispersion was the chief contributor to the stacked dimers. Despite their reliance on electrostatic attraction, the binding energies of the π-hole complexes did not correlate well with the intensity of this hole. A similar contradiction was noted also by Xu and Li [67], in their calculations of RTrH_2_···NH_3_. These authors explained these inconsistencies in terms of the crucial role of orbital interaction and polarization energy.

Recently, the prevalence of the triel bond has been expanded [61] to encompass carbenes and silylenes as electron donors to TrR_3_. The interaction energies of these complexes were surprisingly large, reaching 90 kcal/mol. There were also quite substantial geometric deformations in the monomers caused by the interaction, which lowered the binding energies, which appears to be a common feature of TrBs. Consistant with the aforementioned findings of a poor relationship between binding energy and π-hole intensity, there was little correlation observed here between the electron-withdrawing power of the substituent attached to the Lewis acid and the binding energy. The cooperativity involved in TrBs has been explored, combined with the halogen bond [74], anion–π interactions [58] or the regium bond [68].

Not all noncovalent interactions involve separate molecules. Just as in the case of H-bonds, intramolecular interactions are important in terms of establishing the structure and function of essential molecules such as proteins [75,76]. These internal noncovalent bonds are also involved in supramolecular recognition [77,78]. There has been a respectable amount of study of intramolecular contacts steered by pnicogen [79], tetrel [80,81,82] or chalcogen bonds [78,83,84]. In contrast, however, there is far less information available regarding the intramolecular triel bond (IMTrB). As one of only a few examples, Pla et al. [85] examined a series of naphthyl-bridged amino-borane derivatives and concluded that the naphthyl scaffold exhibits flexibility as a response to B···N interactions. Their NBO analysis revealed a B···N dative bond between an N lone pair and a vacant virtual B orbital. Very recently, the bifurcated TrB in hydrides, fluorides, and chlorides of 1,8-bis(dichloroboryl)naphthalene and 1,2-bis(dichloroboryl)benzene was examined by Grabowski [64]. The anionic structures linked by a BXB bridge (X = H, F, Cl) were characterized as partly covalent. A CSD survey confirmed the presence of similar crystal structures with not only boron but other triel atoms: Al and Ga.

The current work examines the issue of both inter and intramolecular TrBs, and the competition between them. The naphthalene system offers a convenient and well-structured skeleton on which to base this work. A TrF_2_ group is placed on one of the α-positions. On the neighboring C^α^ of the other ring is positioned a NH_2_ nucleophile. These two groups are thus well oriented to engage in an internal Tr···B interaction. Due to its geometry, the TrF_2_ group ought to have a second π-hole that can engage in a second TrB with an approaching nucleophile. The central question is how these two TrBs, one internal and the other external, affect one another. Does the presence of one inhibit the formation of the second, or might the two reinforce one another? Does the intermolecular TrB, free to adopt its optimal geometry with no internal structural restraints, cause the internal TrB to break? How do the two TrBs, either separately or cumulatively, affect the geometry of the TrF_2_ unit, and how does its deformation play into the properties of the two TrBs? In order to address this problem in a broad sense, the Tr atom was varied, from the smallest B, all the way up to Tl. In terms of the approaching external nucleophile, NCH was considered as a weak base with sp hybridation of the N. On the other end of the spectrum, the CN^−^ anion, with its full negative charge, ought to represent a very strong base.

## 2. Results

### 2.1. Monomers

**Figure 1:**
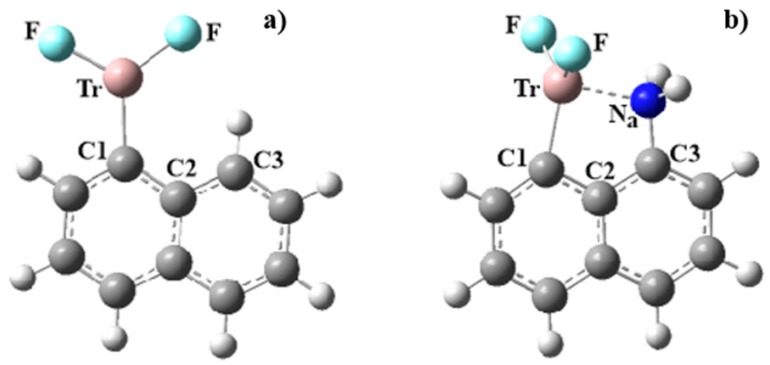
Structure of (**a**) C_10_H_7_TrF_2_ and (**b**) C_10_H_6_NH_2_TrF_2_.

The structure of the naphthyl ring with a TrF_2_ substituent on C^α^ is pictured on the left side of Figure 1, where the TrF_2_ group is itself fully planar and also coplanar with the aromatic system. There is the germ of a CH···F H-bond present, with the H atom attached to C3. As noted in Table S1, the R(H···F) distance varies between 2.2 and 2.5 Å; however, the θ(CH···F) angle deviates by 70°–80° from linearity, so any such HB would be rather weak. Replacing the neighboring C^α^H with a NH_2_ group causes the TrF_2_ to rotate around to become perpendicular with the rings. In so doing, it allows the N lone pair to engage with the vacant p-orbital of the Tr atom. The distance between the Tr and N atoms is less than 2.5 Å in all cases, as may be seen in Table 1. This distance elongates along with a growing Tr atom, but is particularly short for Tr = B at only 1.73 Å. There is strong evidence of a triel bond by way of NBO consideration of charge transfer from N to Tr. The values of E(2) for this transfer are as high as 97 kcal/mol, and diminish along with Tr size down to 39 kcal/mol for Tl. Note that these quantities parallel the Tr···N separations in the preceding column. The B···N interaction is so strong that it is considered a covalent bond via NBO, commensurate with the very short B···N distance. The next column displays the electron density of the Tr···N bond critical point. Like NBO, AIM also assesses a strong B···N bond, bordering on covalent. On the other hand, ρ_BCP_ is curiously low for Al, unlike NBO, which views the Al···N bond as strongest, with the exception of B.

A second measure of an internal Tr···N attraction rests on the bond angles. The attraction between these two atoms ought to bend them toward one another. In particular, the θ(C2C1Tr) and θ(C2C3H) angles in the unsubstituted naphthalene molecules of Figure 1a are both roughly 120°, but the presence of the internal triel bond causes the Tr and N atoms to bend in toward one another when the H is replaced by NH_2_. As shown in Table 1, these inward bends are sizable, as much as 16°. The combined bending is also largest for Tr = B, diminishing as the Tr atom grows in size.

There is significant puckering of both the Tr and N atoms, as evident in the sums of the three angles surrounding them. Full planarity would lead to an angle sum of 360° and a full tetrahedral geometry would lead to a value of 328.5°. While the N atom is close to a tetrahedron, the larger angle sums for Tr are closer to planarity, particularly for the larger Tr atoms. The R(F···H) distances vary from only 2.2 Å for Tl up to 3.2 Å for In, but the very acute θ(NH···F) angles argue against any true HB between the NH_2_ and TrF_2_ groups.

**Figure 2:**
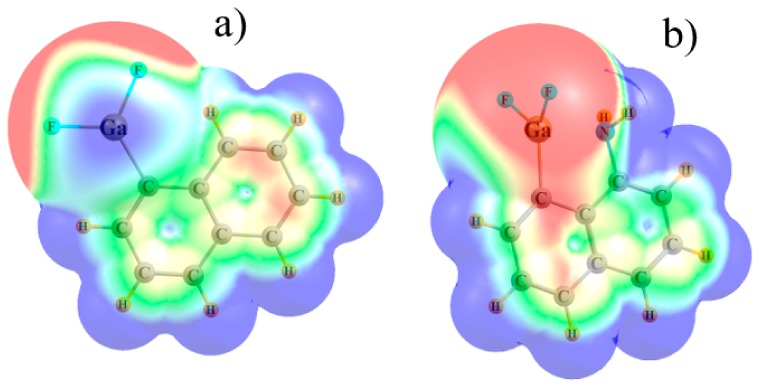
MEPs of (**a**) C_10_H_7_GaF_2_ and (**b**) C_10_H_6_NH_2_GaF_2_. Blue and red regions refer, respectively, to +0.02 and −0.02 au.

The molecular electrostatic potentials surrounding these molecules are represented in Figure 2 for Tr = Ga; the others are all quite similar. There is an intense (blue) pair of equivalent π-holes that lie directly above and below the Tr atom in the unsubstituted C_10_H_7_TrF_2_. The NH_2_ group occupies one of these two π-holes and reduces the intensity of the other, even displacing it slightly toward the peripheral H atoms. The magnitudes of these π-holes are quantified by the value of the maximum of the MEP on the 0.001 au isodensity surface. The values of V_s,max_ reported in Table 2 display some interesting patterns. Considering first the unsubstituted C_10_H_7_TrF_2_ molecules, V_s,max_ follows the order B < Tl < Ga < In < Al, not at all like the order of atom size or electronegativity. Most importantly, in all cases this quantity is reduced by the triel bond in C_10_H_6_NH_2_TrF_2_. This decrease is summarized in the last column of Table 2 and varies between 9 kcal/mol for B up to as much as 32 kcal/mol for Al.

### 2.2. Complexes with NCH

**Figure 3:**
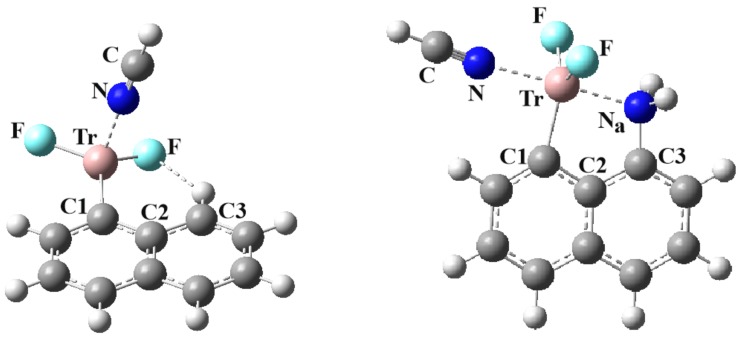
Optimized geometries of complexes of NCH with C_10_H_7_TrF_2_ (left) and C_10_H_6_NH_2_BF_2_ (right) at MP2/aug-cc-pVDZ level.

The geometries obtained when an NCH molecule is added to the various monomers are illustrated in Figure 3. Whether unsubstituted or substituted, the NCH base adds to the site of the π-hole on the Tr atom. An important exception arises for Tr = B, where a triel bond is not formed. The NCH molecule swings around so as to engage in a NCH···F HB with one of the two BF_2_ fluorine atoms. This failure to form a triel bond with the B is not entirely surprising in view of the shallow π-hole for C_10_H_7_BF_2_ and C_10_H_6_NH_2_BF_2_, listed in Table 2.

Figure 3 also presents a difference between the structure of the H- and NH_2_-substituted naphthalenes. In keeping with the geometries of the monomers, in the unsubstituted case the TrF_2_ group is basically coplanar with the naphthalene, so that the Tr p-orbital can conjugate with the naphthyl aromatic π-system, whereas the group rotates around by roughly 90° so that this same orbital can interact with the N lone pair after NH_2_ substitution. In the first case, then, the nucleophile approaches from above the naphthyl ring plane, whereas the approach in the latter case is nearly coplanar.

The salient geometrical parameters of the Tr-bonded complexes with the NCH contained in Table 3 show first that the intermolecular R(Tr···N) distance elongates as the Tr atom grows in size. The θ(C-N⋅⋅⋅Tr) angle is roughly linear, while the θ(C1-Tr···N) angle shows the NCH approaching nearly perpendicular to the C1-T axis, i.e., the Tr π-hole. The TrF_2_ group remains close to planar, with Σθ_Tr_ quantities all close to 360°.

The corresponding geometrical data for the substituted naphthalenes are supplied in Table 4. The weakening of the π-hole by the NH_2_ group lengthens all of the R(Tr···N) distances by some 0.1–0.3 Å. There is a mutual negative cooperativity in that the formation of the external triel bond also lengthens the internal bond, by 0.04–0.06 Å. The NCH approaches at a nearly perpendicular angle to the C1-Tr bond, between 91° and 99°. However, as the Tr atom grows larger there is an increasing tendency for the NCH molecule to pivot around its N atom, which decreases the θ(C-N⋅⋅⋅Tr) angle. In fact, for Tr = Tl, the NCH molecule bends around so that it is essentially parallel to one of the two Tl-F bonds. Another manifestation of the ability of the external TrB to weaken the internal one can be seen in the bending angles. Whereas the Tr atom is bent in toward the N by some 5°–16° to form the Tr···N bond in the monomer (Table 1), the amount of this bending is reduced by the external Tr···NCH bond in Table 4, down to only 3°–11°. As in the other cases, the TrF_2_ unit retains its planarity, with Σθ_Tr_ equal to 360°, and the internal NH_2_ unit is pyramidal. There is a closer proximity of the NH protons to the TrF fluorines caused by the external NCH.

The weakening effect of the internal Tr···N interaction on the triel bond with NCH is obvious from a comparison of the top and bottom sections of Table 5. E_b_ refers to the binding energy of the complex relative to fully optimized monomers, while the interaction energy in the next column considers the monomers in the geometries they adopt within the complex. This weakening is greatest for Al at 10.0 kcal/mol, and diminishes along with the Tr atom size down to only 0.5 kcal/mol for Tl. Note again that this weakening is so important for B that there is no triel-bonded complex with NCH. The difference between E_b_ and E_int_ is equal to the deformation energy required for the two monomers to change their geometry to that within the complex, listed in Table 5 as E_def_. This quantity is largest for the smaller Tr atoms, decreasing from 4 kcal/mol for Al down to less than 1 kcal/mol for Tl. The fourth column of Table 5 allows an evaluation of the effects of level of theory on the computed data. The interaction energies, calculated with the CCSD(T) treatment of electron correlation, are quite similar to those obtained by MP2, verifying the accuracy of the calculations.

The last column of Table 5 contains the value of the density at the Tr···N bond critical point. While these quantities are not linearly related to the bond strength, they clearly document the weakening of each triel bond by the presence of the internal Tr···N interaction. The AIM molecular diagrams in Figure S1 show that the Tr···N bond is the only intermolecular interaction present for the unsubstituted naphthalene derivatives. However, there are also assorted secondary interactions present for HCN···C_10_H_6_NH_2_TrF_2_. Most important are a CH···N HB and a C···F tetrel bond for Tl, which help to explain its distorted geometry.

### 2.3. Complexes with NC^−^ Anion

**Figure 4:**
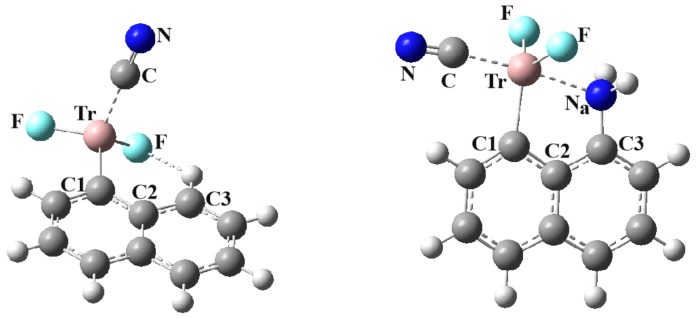
Optimized geometries of complexes of NC^-^ with C_10_H_7_TrF_2_ (left) and C_10_H_6_NH_2_BF_2_ (right) at MP2/aug-cc-pVDZ level.

The negative charge on CN^−^ ought to make it a much stronger nucleophile. As exhibited in Figure 4, it is the C atom that approaches Tr, rather than the N. Otherwise, the superficial aspects of the structure of this anion, with the naphthalene derivatives in Figure 4, look very much like those for neutral HCN in Figure 3. The greater nucleophilicity of this anion allows the formation of a B-triel bond to N, which did not occur for the neutral HCN. This is quite a short bond, with R(B···C) = 1.643 Å, as indicated in Table 6 for the unsubstituted naphthalenes. The other systems also exhibit a contraction of the triel bond length relative to NCH, varying from −0.08 Å for Al up to −0.34 Å for Tl. The anion also enlarges the θ (C1-Tr···N) angle by 9°–12° for most of these systems, but by 25° for Tl. This change accompanies a further pyramidalization of the TrF_2_ group, as the sum of the three relevant angles drops from almost 360° to about 330°.

Just as in the unsubstituted derivatives, here again the triel bonds of C_10_H_6_NH_2_TrF_2_ shrink upon going from NCH to NC^−^. A comparison of Table 4 with Table 7 shows a contraction that varies from −0.15 for Al up to −0.58 Å for Tl. Along with this contraction is associated a concomitant lengthening of the internal Tr···N_a_ bond length, which lies in the 0.19–0.85 Å range. The θ(NC···Tr) angle is very close to linear, and the θ(C1-Tr···C) angle increases as well, to more than 90° in all cases. The latter increase is consistent with the pyramidalization of the Tr atom, wherein the sum of the three angles is some 8°−25° less than 360°. Due to the strong external TrB to CN^−^, there is additional geometric evidence of the weakening of the internal Tr···N bond. In the previous systems, whether the monomer or the complex with NCH, the substitution of an NH_2_ group on the naphthalene caused the Tr and N atoms to bend in toward one another. However, there is very little bending of this sort when the Tr is engaged in strong external TrB to CN^−^. Indeed, some of the changes in θ(C2C1Tr) and θ(C2C3N_a_) in Table 7 are positive, suggesting that the Tr and N_a_ atoms actually move away from one another, even if only by a small amount.

The energetic aspects of this anion-induced bond strengthening are clear from a comparison of Table 5 and Table 8. Aside from the ability of NC^−^ to engage in a triel bond with B, which NCH could not, there is a strong enhancement of the binding energies. Both E_b_ and E_int_ are magnified by a factor between 3 and 10. The largest binding energy of 69 kcal/mol occurs for the unsubstituted NC^−^⋅⋅⋅C_10_H_7_AlF_2_. Even with the competition of an internal triel bond, the substituted naphthalenes see binding energies as high as 52 kcal/mol, with a minimum of 21 kcal/mol for NC^−^···C_10_H_6_NH_2_BF_2_. The anion-induced interactions are also reflected in the larger bond critical point densities in the last column of Table 5 and Table 8. As in the NCH cases, the MP2 interaction energies mimic the same quantities calculated with CCSD(T).

Whereas the binding and interaction energies of NCH were fairly close to one another, there is a large discrepancy for the anion. The much larger interaction energies are a result of the large-scale geometry changes caused by the stronger interaction. Of particular note in this regard is the large distortion from planarity of each TrF_2_ group, as pointed out by the large deviations from 360° in the Σθ_Tr_ quantity. Another important factor is the stretch of each internal Tr···N triel bond.

As was the case for the neutral ligands, the complexes of the unsubstituted naphthalenes with NC^−^ are also stabilized by a sole intermolecular Tr···C bond, within the AIM context, as may be seen in Figure S2. Consistent with the HCN situation, there are additional secondary interactions for C_10_H_6_NH_2_TrF_2_. This noncovalent bond occurs only for the three lightest Tr atoms, and can be characterized as a very weak CH···C HB. It is interesting to observe that, in the case of the strong CN^−^ Lewis base, there is evidence that the internal Tr···B bond weakens to the point of vanishing, as may be seen by the lack of a pertinent bond path in Figure S2, which is replaced in certain cases by one or more NH···F HBs. This internal bond weakening/disappearance explains the lack of bending of the Tr and N_a_ atoms toward one another, alluded to above.

Finally, the total interaction energy may be partitioned into separate contributions. The results of such a partitioning are delineated in Tables S2 and S3 for the HCN and NC^−^ ligands, respectively. Similar to many other related noncovalent bonds, electrostatics account for slightly more than half of the total interaction energy. The orbital interaction term is somewhat smaller. Its percentage contribution is some 30–40% for HCN. The same percentage is also true for the anion, with the exception of Tr = B, where E_oi_ rises to roughly 50%. Dispersion makes a negligible contribution to these complexes, in the range 1–2% for the anion, and slightly larger for HCN.

## 3. Discussion

The Cambridge Structural Database (CSD) [86] provides some experimental context for the bonding schemes studied here. First is the question of systems containing an internal triel bond of the sort depicted in Figure 1. Taking the naphthalene unit as a building block, the CSD was searched for systems wherein a Tr atom was located on one C^α^ atom and an N on the neighboring C^α^, as in Figure 1. The definition of an internal triel bond is based on the R(Tr··N) distance being less than the sum of vdW radii of the Tr and N atoms. The minimum for this range of distances is an arbitrary one, but ought to avoid a purely covalent bond. Each row of Table S4 refers to a particular percentage of the sum of covalent atomic radii, varying from 110% to 140%. It may be seen that the bulk of observations arise for Tr = B where there are between nine and 13 cases, depending upon the chosen minimum distance. There are fewer observations for the heavier Tr atoms, most of which are only slightly longer than the covalent bond length. This finding is consistent with the computed data in Table 1 that suggest the internal B···N bond is the shortest and strongest of those considered, hovering around the range of a covalent bond. Table 1 documents the manner in which the Tr and N atoms bend toward one another as a result of the internal triel bond. This sort of bending is plainly seen in a number of crystal structures, examples of which are provided in Table S5. The θ(C2C1Tr) and θ(C2C3H) angles are 120°–125° and 120°, respectively, when the N atom is absent. But these angles are quite a bit smaller in those cases where a N substituent is present on the naphthalene system, particularly when the R(Tr···N) distance is short. It might be noted, finally, that the CSD provides some evidence in Table S5 wherein the Tr atom participates not only in this internal Tr···N bond, but also in a second Tr bond of the sort depicted in Figure 3 and Figure 4. It is not only B for which this is true but also Al, In, and Tl.

The findings presented above can be placed in the context of several earlier sets of calculations. For example, an earlier study [87] of an intramolecular B···N interaction in various naphthyl-bridged amino–borane compounds calculated NBO E(2) energies for LP(N)→LV(B) transfer that reached 164.2 kcal/mol, although this quantity is much smaller, in the range of 4–10 kcal/mol, for the other systems. The first large value is consistent with our own finding by NBO of a covalent B···N bond, with LP(N)→LP*(Tr) values between 39 and 97 kcal/mol.

Our own earlier study of a triel bond pairing TrR_3_ with pyrazine [73] applied the same level of theory as here, but involved a different base. In order to facilitate some comparison, it could be considered that the GaH_3_ molecule has a π-hole very similar in magnitude to that of C_10_H_7_GaF_2_. Conflating the data for the different bases, the order of interaction energy diminishes as CN^−^ > pyrazine > NCH. As in the current work, the earlier calculations also indicated a non-negligible role of deformation energy, which was largest for B. In keeping with its placement between NCH and CN^−^ in terms of binding energy, pyrazine also is associated with deformation energies between these two extremes. Energy decompositions are also similar for the various bases, in that the contribution of electrostatic and orbital contributions are nearly equal for B, while the percentage contribution of electrostatics grows along with a decrease in the orbital interaction component for the other Tr atoms.

Grabowski’s [64] systems encompassed dichloroboryl derivatives of naphthalene and benzene, wherein the Tr atoms situated on the same molecule were linked by H, F or Cl anions, somewhat akin to our own CN^−^ nucleophile. As such, each anion was held by what might be considered a pair of Tr bonds to B, which might explain the large interaction energies between 104 and 161 kcal/mol. But nonetheless, these systems also showed a large influence of deformation energies, as high as 50 kcal/mol. Despite some difference in structural form, these systems displayed a pattern of energy decomposition components similar to those observed here. It might also be noted that the AIM BCP densities for some of the stronger complexes exceeded 0.1 au, suggesting covalency. Similarly, large ρ_BCP_ values were observed here for complexes of the CN^−^ anion with B-derivatives. Earlier calculations [86] verify the ability of a Tr atom to engage in a pair of triel bonds simultaneously, one on each side of a planar TrF_3_ molecule, and echo the weakening the second bond causes to the first, as was observed here.

## 4. Computational Methods

The geometries of the naphthalene derivatives C_10_H_7_TrF_2_ and C_10_H_6_NH_2_TrF_2_ (Tr = B, Al, Ga, In, Tl) and their complexes with HCN and CN^−^ were all optimized at the MP2 level of theory in combination with the aug-cc-pVDZ basis set [88,89]. For the purpose of including relativistic effects for heavy In and Tl atoms, aug-cc-pVDZ-PP pseudopotentials were incorporated [90,91,92,93,94,95]. Frequency calculations were carried out at the same level to verify that the obtained geometries are true minima with no imaginary frequencies. The energies were recalculated at the CCSD(T)/aug-cc-pVDZ level (making use of MP2 geometries) to verify their accuracy [96,97,98]. The interaction energy (E_int_) is defined here as the difference between the energy of the complex and the sum of monomers, with the latter in the geometry they adopt within the complex. The binding energy (E_b_) takes as its reference the optimized pre-deformed isolated monomers. Thus, the difference between interaction and binding energy is the deformation energy E_def_. All energies were corrected for basis set superposition error (BSSE) via the counterpoise protocol [99]. Computations were performed using the Gaussian 16 suite of codes [100].

The molecular electrostatic potentials of isolated monomers were analyzed through the MultiWFN and WFA-SAS programs in order to obtain V_s,max_ and V_s,min_ values as well as electrostatic potential maps on the 0.001 au electronic isodensity surface [101,102,103,104]. The quantum theory of atoms in molecules (QTAIM) was employed to locate and characterize bond critical points (BCPs) in interacting systems using AIMAll software [105]. The NBO method was applied (GenNBO 6.0 program) to compute the orbital–orbital interactions using the wavefunction generated at the DFT level for the MP2 optimized geometries [106,107,108,109]. The decomposition of interaction energies was applied to partition E_int_ into the following components: electrostatic, orbital interaction, dispersion and repulsive forces. The EDA scheme embedded in the ADF software at the BLYP/ZORA/TZ2P level was used for this purpose [110,111,112]. Finally, the CSD survey [86] with specified bond distances criteria was performed using the ConQuest program [113] so as to identify experimental crystal structures with certain bonding patterns related to those found via calculations.

## 5. Conclusions

In summary, the TrF_2_ group is capable of engaging in a strong intramolecular TrB with a neighboring NH_2_ group. When both groups are located on the α positions of a naphthalene unit, this TrB completes a five-membered TrCCCN ring. This TrB reduces the intensity of the π-hole on the Tr atom, decreasing its ability to engage in a second external TrB. This weakening is true for both ends of the spectrum: the weak NCH base and the much stronger nucleophile anionic CN^−^. The external TrB is weakened by a factor of about two for the smaller Tr atoms but is less severe for the larger Tl. The intermolecular TrB can be quite strong nonetheless; it varies from a minimum of 8 kcal/mol for the weak NCH base, up to as much as 70 kcal/mol for CN^−^ anion. This anticooperative effect is mutual in a sense. The appearance of an external TrB to a strong base like CN^−^ lessens the ability of the Tr to engage in an internal TrB, to the point where such an intramolecular TrB becomes questionable.

## Figures and Tables

**Table 1:** Structural parameters (distances in Å, angles in degrees), and the NBO value of E(2) for transfer from N to Tr (kcal/mol), and density at Tr···N AIM bond critical point (au), of C_10_H_6_NH_2_TrF_2_ calculated at the MP2/aug-cc-pVDZ level of theory.

	R(Tr⋅⋅⋅N_a_)	E(2)	ρ_BCP_	Δθ(C2C1Tr) ^a^	Δθ(C2C3N_a_) ^a^	Σθ_Tr_^b^	Σθ_N_^c^	R(F⋅⋅⋅H_N_)	θ(NH⋯F)
C_10_H_6_NH_2_BF_2_	1.727	^d^	0.098	−15.6	−9.4	346.8	332.4	2.435	81
C_10_H_6_NH_2_AlF_2_	2.058	97.3	0.048	−15.8	−3.6	355.2	327.9	2.875	83
C_10_H_6_NH_2_GaF_2_	2.103	58.6	0.070	−13.5	−3.5	356.4	329.7	2.919	85
C_10_H_6_NH_2_InF_2_	2.321	51.8	0.057	−9.1	−1.6	358.8	327.8	3.216	87
C_10_H_6_NH_2_TlF_2_	2.443	39.1	0.052	−4.9	−1.8	359.9	331.8	2.243	89

^a^ change in angle caused by replacement of H by NH_2_ group. Negative value signifies bend of Tr and N toward one another.^b^ θ(F-Tr-F) + θ(F-Tr-C1) + θ(F-Tr-C1). ^c^ θ(H-N-H) + θ(H-N-C3) + θ(H-N-C3). ^d^ NBO considers the B⋯N interaction to be a covalent bond.

**Table 2:** Maxima in MEP (kcal/mol) of isolated C_10_H_7_TrF_2_ and C_10_H_6_NH_2_TrF_2_ monomers calculated at the MP2/aug-cc-pVDZ level of theory.

Isolated Molecule	V_S,max_	Isolated Molecule	V_S,max_	Δ
C_10_H_7_BF_2_	32.0	C_10_H_6_NH_2_BF_2_	22.9	−9.1
C_10_H_7_AlF_2_	78.3	C_10_H_6_NH_2_AlF_2_	45.8	−32.5
C_10_H_7_GaF_2_	65.3	C_10_H_6_NH_2_GaF_2_	39.2	−26.1
C_10_H_7_InF_2_	70.1	C_10_H_6_NH_2_InF_2_	50.7	−19.4
C_10_H_7_TlF_2_	52.9	C_10_H_6_NH_2_TlF_2_	40.0	−12.9

**Table 3:** Structural parameters (distances in Å, angles in degrees) in complexes of C_10_H_7_TrF_2_ with NCH calculated at the MP2/aug-cc-pVDZ level of theory.

	R(Tr⋅⋅⋅N)	θ(C-N⋅⋅⋅Tr)	θ(C1-Tr···N)	Σθ_Tr_^a^	R(CH···F)
HCN⋅⋅⋅C_10_H_7_AlF_2_	2.101	175.8	99.1	354.4	2.426
HCN⋅⋅⋅C_10_H_7_GaF_2_	2.178	179.1	103.2	355.4	2.405
HCN⋅⋅⋅C_10_H_7_InF_2_	2.399	176.0	102.3	357.4	2.471
HCN⋅⋅⋅C_10_H_7_TlF_2_	2.541	168.1	101.4	359.1	2.335

^a^ θ(F-Tr-F) + θ(F-Tr-C1) + θ(F-Tr-C1).

**Table 4:** Structural parameters (distances in Å, angles in degrees) in complexes of C_10_H_6_NH_2_TrF_2_ with NCH calculated at the MP2/aug-cc-pVDZ level.

	R(N⋅⋅⋅Tr)	R(Tr⋅⋅⋅N_a_)	θ(C-N⋅⋅⋅Tr)	θ(C1-Tr···N)	Δθ(C2C1Tr) ^a^	Δθ(C2C3N_a_) ^a^	Σθ_Tr_	Σθ_N_	R(F⋅⋅⋅H_N_)
HCN···C_10_H_6_NH_2_AlF_2_	2.221	2.115	173.2	93.6	−11.1	−3.9	359.9	329.7	2.656
HCN···C_10_H_6_NH_2_GaF_2_	2.317	2.162	166.1	96.8	−9.8	−3.4	359.9	331.5	2.727
HCN···C_10_H_6_NH_2_InF_2_	2.489	2.369	162.7	99.2	−6.7	−1.6	360.0	329.3	3.050
HCN···C_10_H_6_NH_2_TlF_2_	2.813	2.485	94.0	91.0	−2.9	−1.7	359.1	332.2	2.175

^a^ change in angle caused by replacement of H by NH_2_ group. Negative value signifies bend of Tr and N toward one another.

**Table 5:** Binding, interaction, and deformation energies (kcal/mol) and density at the Tr···N bond critical point (au) of naphthalene derivatives complexed with NCH corrected for basis set superposition error (BSSE).

	E_b_	E_int_	E_def_	E_int_ (CCSD(T))	ρ_BCP_
HCN⋅⋅⋅C_10_H_7_AlF_2_	−18.08	−22.36	4.28	−21.26	0.035
HCN⋅⋅⋅C_10_H_7_GaF_2_	−13.60	−17.01	3.41	−15.86	0.049
HCN⋅⋅⋅C_10_H_7_InF_2_	−13.41	−15.11	1.70	−14.23	0.040
HCN⋅⋅⋅C_10_H_7_TlF_2_	−8.85	−9.57	0.72	−8.51	0.035
HCN···C_10_H_6_NH_2_AlF_2_	−8.10	−12.25	4.15	−11.41	0.026
HCN···C_10_H_6_NH_2_GaF_2_	−6.21	−9.09	2.88	−8.20	0.035
HCN···C_10_H_6_NH_2_InF_2_	−9.28	−10.62	1.34	−9.94	0.032
HCN···C_10_H_6_NH_2_TlF_2_	−8.34	−8.93	0.59	−8.58	0.020

**Table 6:** Structural parameters (distances in Å, angles in degrees) in complexes of C_10_H_7_TrF_2_ with NC^−^ calculated at the MP2/aug-cc-pVDZ level of theory.

	R(Tr⋅⋅⋅C)	θ(N-C⋅⋅⋅Tr)	θ(C1-Tr···C)	Σθ_Tr_	R(CH···F)
NC^−^⋅⋅⋅C_10_H_7_BF_2_	1.643	178.4	110.0	330.7	2.374
NC^−^⋅⋅⋅C_10_H_7_AlF_2_	2.025	178.8	110.0	332.9	2.482
NC^−^⋅⋅⋅C_10_H_7_GaF_2_	2.015	178.0	115.1	329.8	2.447
NC^−^⋅⋅⋅C_10_H_7_InF_2_	2.204	176.0	111.3	330.3	2.217
NC^−^⋅⋅⋅C_10_H_7_TlF_2_	2.205	175.8	126.0	323.1	2.116

**Table 7:** Structural parameters (distances in Å, angles in degrees) in complexes of C_10_H_6_NH_2_TrF_2_ with NC^−^ calculated at the MP2/aug-cc-pVDZ level of theory.

	R(Tr⋅⋅⋅C)	R(Tr⋅⋅⋅N_a_)	θ(N-C⋅⋅⋅Tr)	θ(C1-Tr···C)	Δθ(C2C1Tr)^a^	Δθ(C2C3N_a_)^a^	Σθ_Tr_	Σθ_N_	R(F⋅⋅⋅H_N_)
NC^−^⋅⋅⋅C_10_H_9_NBF_2_	1.658	2.843	179.2	109.3	4.8	−0.1	337.1	336.8	2.828
NC^−^⋅⋅⋅C_10_H_9_NAlF_2_	2.067	2.302	174.5	97.6	−6.8	−3.8	352.3	335.4	2.446
NC^−^⋅⋅⋅C_10_H_9_NGaF_2_	2.017	3.014	177.1	109.4	6.6	1.8	335.4	341.9	1.791
NC^−^⋅⋅⋅C_10_H_9_NInF_2_	2.222	2.803	174.8	105.3	0.8	0.0	340.6	338.1	1.864
NC^−^⋅⋅⋅C_10_H_9_NTlF_2_	2.236	2.987	173.1	112.4	4.1	1.9	334.6	341.5	1.711

^a^ change in angle caused by the replacement of H by the NH_2_ group. Negative value signifies bend of Tr and N toward one another.

**Table 8:** Binding, interaction and deformation energies (kcal/mol) and density at Tr···C bond critical point (au) of naphthalene derivatives complexed with NC^−^ corrected for BSSE.

	E_b_	E_int_	E_def_	E_int_ (CCSD(T))	ρ_BCP_
NC^−^⋅⋅⋅C_10_H_7_BF_2_	−48.22	−85.31	37.09	−82.05	0.133
NC^−^⋅⋅⋅C_10_H_7_AlF_2_	−68.96	−88.08	19.12	−86.61	0.059
NC^−^⋅⋅⋅C_10_H_7_GaF_2_	−65.75	−86.32	20.57	−84.53	0.092
NC^−^⋅⋅⋅C_10_H_7_InF_2_	−65.37	−83.68	18.31	−82.19	0.079
NC^−^⋅⋅⋅C_10_H_7_TlF_2_	−56.56	−76.72	20.16	−74.59	0.092
NC^−^···C_10_H_6_NH_2_BF_2_	−21.26	−78.98	57.72	−76.10	0.128
NC^−^···C_10_H_6_NH_2_AlF_2_	−43.54	−67.21	23.67	−65.93	0.055
NC^−^···C_10_H_6_NH_2_GaF_2_	−46.03	−90.11	44.08	−88.55	0.092
NC^−^···C_10_H_6_NH_2_InF_2_	−51.72	−77.54	25.82	−76.19	0.076
NC^−^···C_10_H_6_NH_2_TlF_2_	−49.16	−72.50	23.34	−70.79	0.086

## Supplementary Materials

The following are available online at https://www.mdpi.com/1420-3049/25/3/635/s1, Figure S1: AIM molecular diagrams of complexes of naphthalene derivatives and HCN. Small green dots refer to bond critical points (BCP), labeled with the value of the density at that point (au). The level of calculations is MP2/aug-cc-pVDZ., Figure S2: AIM molecular diagrams of complexes of naphthalene derivatives and NC^−^. Small green dots refer to bond critical points (BCP), labeled with the value of the density at that point (au). The level of calculations is MP2/aug-cc-pVDZ., Table S1: Structural parameters (distances in Å, angles in degrees) of C_10_H_7_TrF_2_ calculated at the MP2/aug-cc-pVDZ level of theory., Table S2: EDA/BLYP/ZORA/TZ2P decomposition of the total DFT-D interaction energy with HCN into Pauli repulsion (E_Pauli_), electrostatic (E_elec_) and orbital (E_oi_) interactions, and dispersion correction (E_disp_). All in kcal mol^−1^., Table S3: EDA/BLYP/ZORA/TZ2P decomposition of the total DFT-D interaction energy with NC^−^ anion into Pauli repulsion (E_Pauli_), electrostatic (E_elec_) and orbital (E_oi_) interactions, and dispersion correction (E_disp_). All in kcal mol^−1^., Table S4: Number of cases identified in the CSD database for which the internal T(Tr···N) distance lies between the indicated percentage of the sum of covalent radii with an upper limit of the sum of vdW radii., Table S5: Examples drawn from the CSD database [taken from ref. 111]. Distances in Å, angles in degs.
