# Supplementary material for: Competition between Intra and Intermolecular Triel Bonds. Complexes between Naphthalene Derivatives and Neutral or Anionic Lewis Bases
Source: Molecules. 2020 Feb 1;25(3):635. doi: 10.3390/molecules25030635 (PMC7037318; doi:10.3390/molecules25030635)
Supplement: Supplementary file 1 [file molecules-25-00635-s001.pdf]

Competition between Intra and Intermolecular Triel Bonds. Complexes between Naphthalene Derivatives and Neutral or Anionic Lewis Bases

Wiktor Zierkiewicz,*[1] Mariusz Michalczyk, [1] and Steve Scheiner*[2]

[1] Faculty of Chemistry, Wrocław University of Science and Technology, Wybrzeże Wyspiańskiego 27, 50-370 Wrocław, Poland

[2] Department of Chemistry and Biochemistry, Utah State University Logan, Utah 84322-0300, United States

Table S1. Structural parameters (distances in Å, angles in degrees) of $C_{10}H_7TrF_2$ calculated at the MP2/aug-cc-pVDZ level of theory.

	R(F···H)	θ(CH···F)
$C_{10}H_7BF_2$	2.191	101
$C_{10}H_7AlF_2$	2.399	110
$C_{10}H_7GaF_2$	2.387	110
$C_{10}H_7InF_2$	2.480	111
$C_{10}H_7TlF_2$	2.317	108

Fig S1. AIM molecular diagrams of complexes of naphthalene derivatives and HCN. Small green dots refer to bond critical points (BCP), labeled with the value of the density at that point (au). The level of calculations is MP2/aug-cc-pVDZ.

$HCN\cdots C_{10}H_7BF_2$	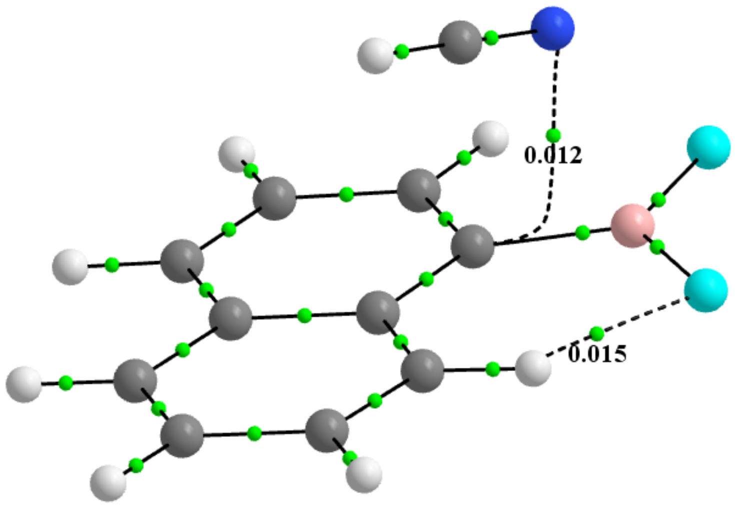
$HCN\cdots C_{10}H_7AlF_2$	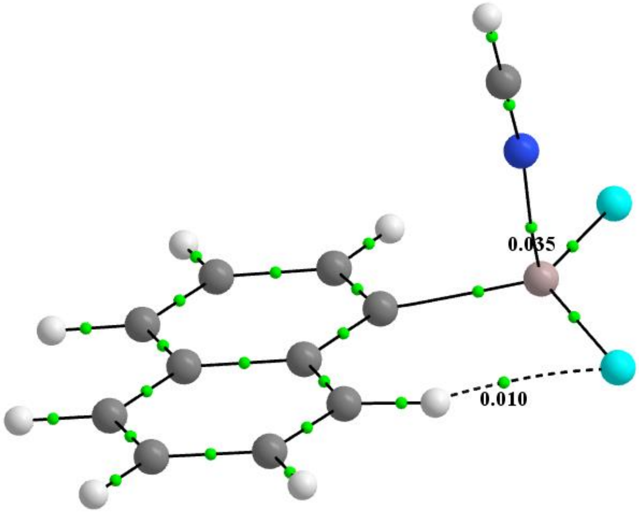
$HCN\cdots C_{10}H_7GaF_2$	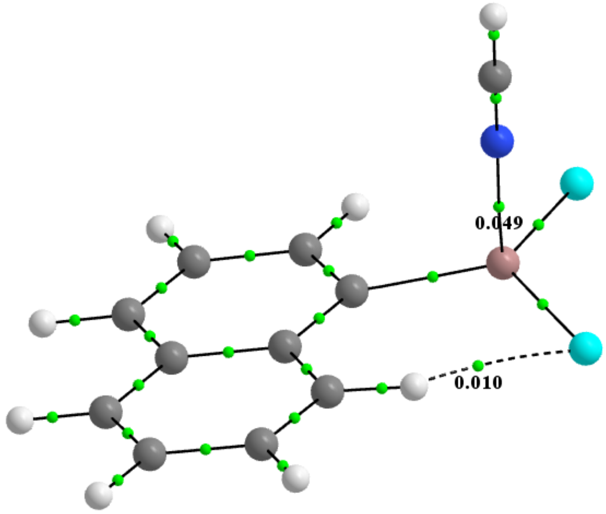

HCN···$C_{10}H_7InF_2$	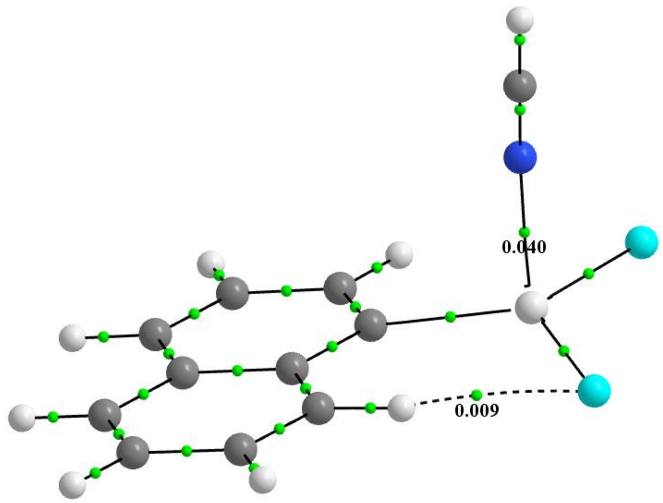
HCN···$C_{10}H_7TlF_2$	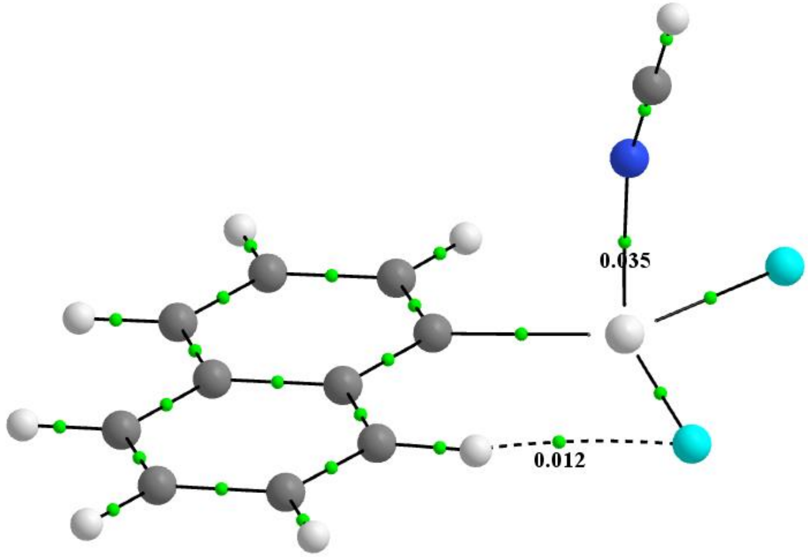

$HCN \cdots C_{10}H_6NH_2AlF_2$	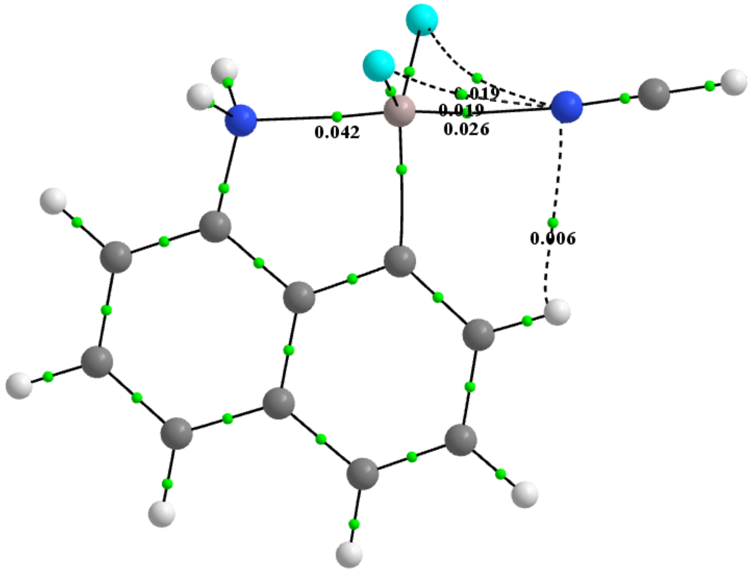
$HCN \cdots C_{10}H_6NH_2GaF_2$	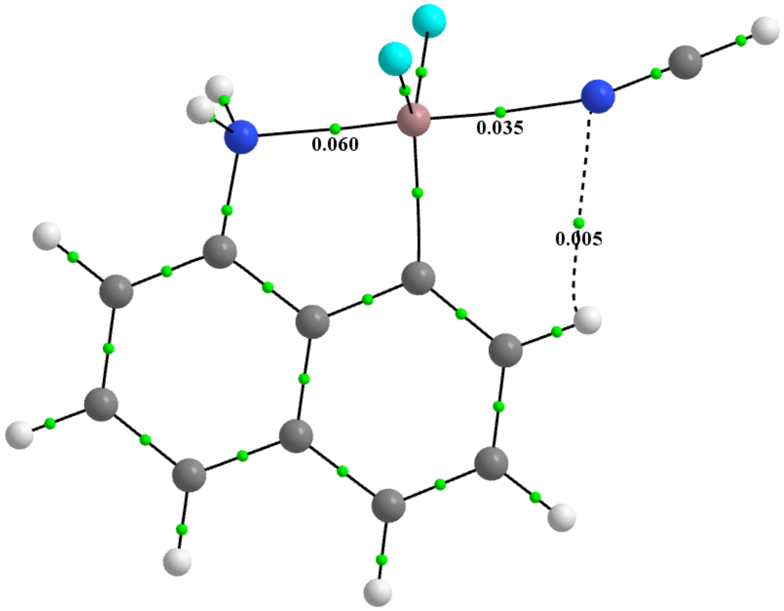

HCN···$C_{10}H_6NH_2InF_2$	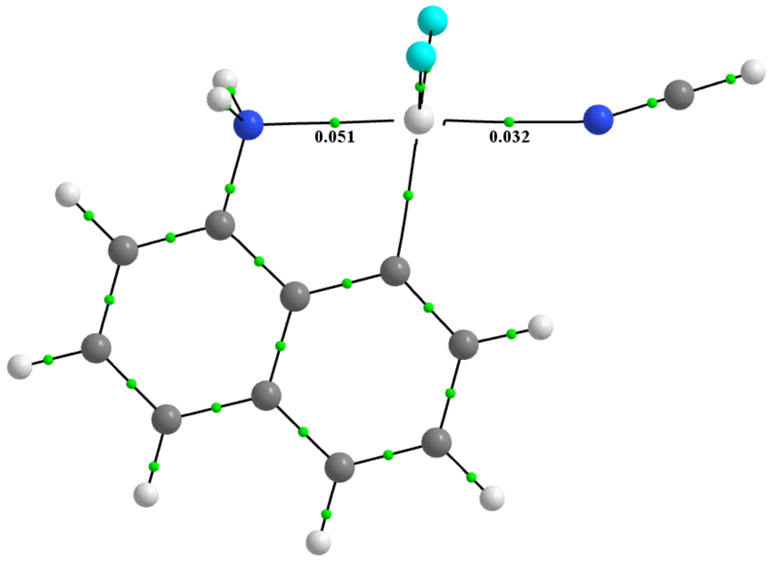
HCN···$C_{10}H_6NH_2TlF_2$	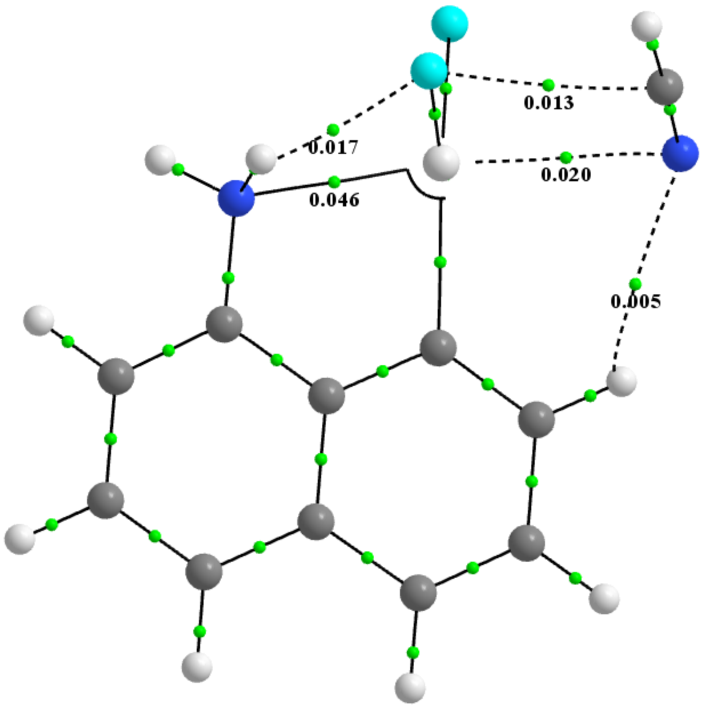

Fig S2. AIM molecular diagrams of complexes of naphthalene derivatives and NC^-. Small green dots refer to bond critical points (BCP), labeled with the value of the density at that point (au). The level of calculations is MP2/aug-cc-pVDZ.

$NC^{-}\cdots C_{10}H_7BF_2$	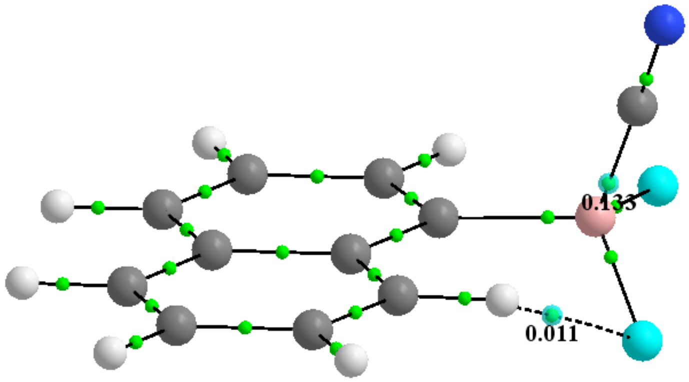
$NC^{-}\cdots C_{10}H_7AlF_2$	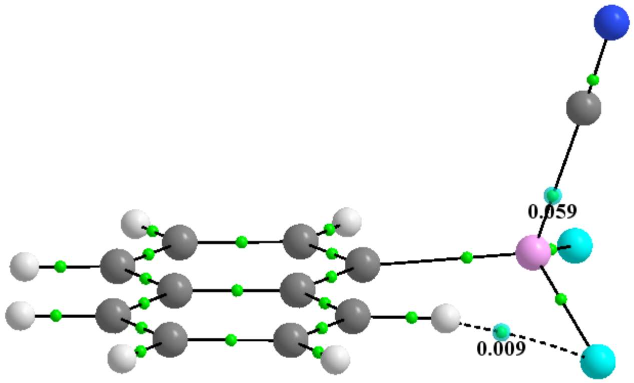
$NC^{-}\cdots C_{10}H_7GaF_2$	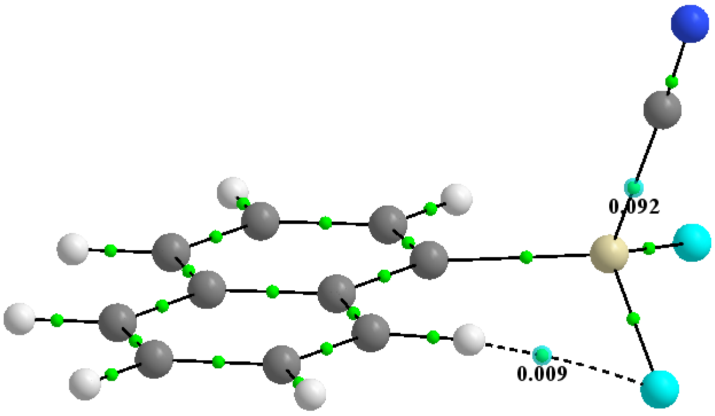

$NC^{-}\cdots C_{10}H_7InF_2$	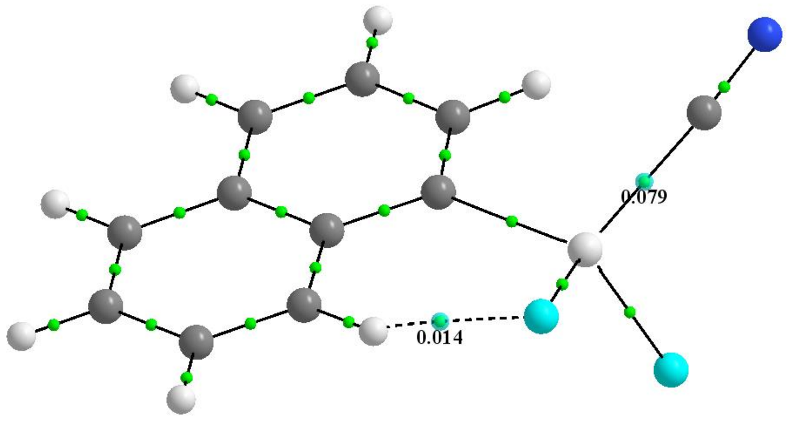
$NC^{-}\cdots C_{10}H_7TlF_2$	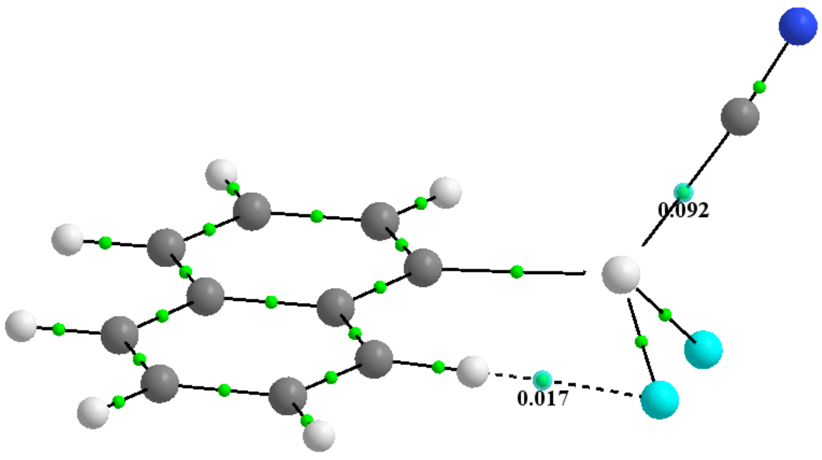
$NC^{-}\cdots C_{10}H_6NH_2BF_2$	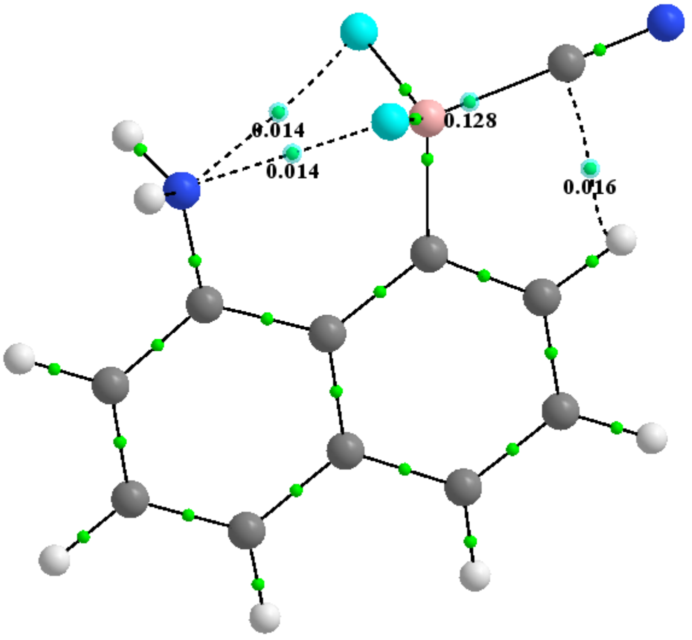

$NC^{-}\cdots C_{10}H_6NH_2AlF_2$	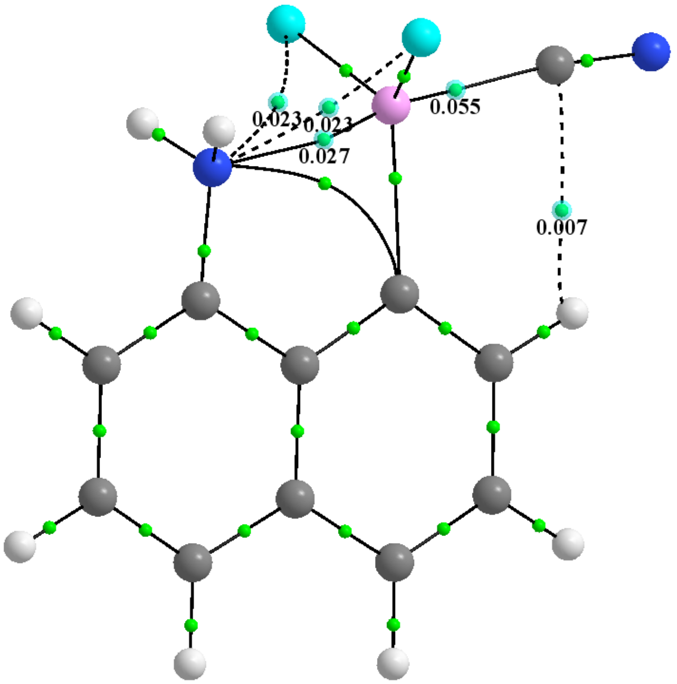
$NC^{-}\cdots C_{10}H_6NH_2GaF_2$	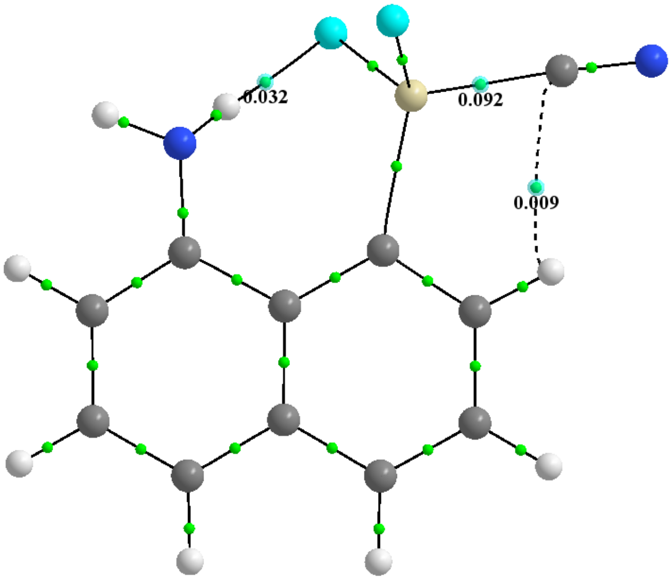

$NC^{-}\cdots C_{10}H_6NH_2InF_2$	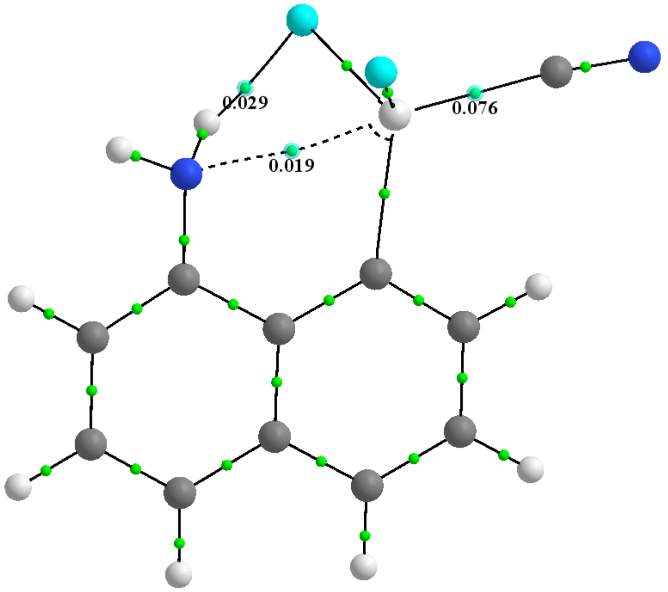
$NC^{-}\cdots C_{10}H_6NH_2TlF_2$	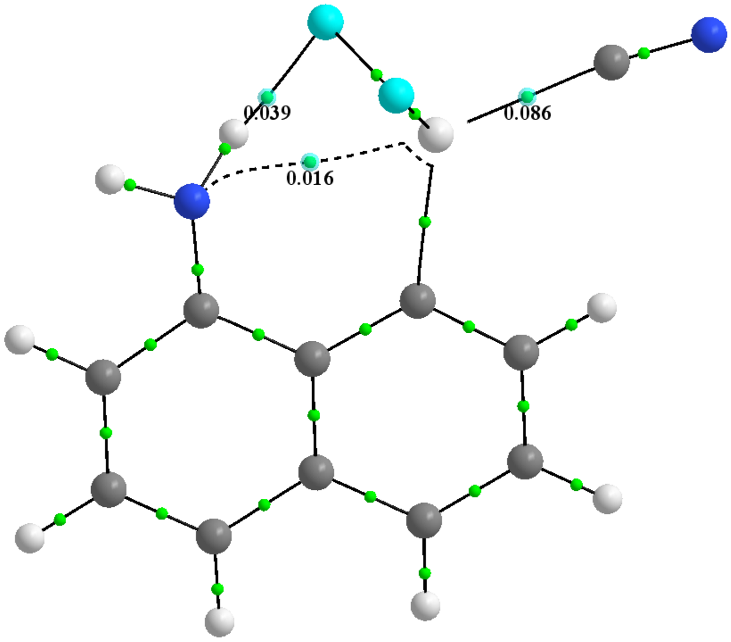

TABLE S2. EDA/BLYP/ZORA/TZ2P decomposition of the total DFT-D interaction energy with HCN into Pauli repulsion (E_{Pauli}), electrostatic (E_{elec}) and orbital (E_{oi}) interactions, and dispersion correction (E_{disp}). All in kcal mol^{-1}.

	total	E_{Pauli}	E_{elec}	%	E_{oi}	%	E_{disp}	%
$HCN\cdots C_{10}H_7AlF_2$	-20.60	47.94	-38.22	56	-27.28	40	-3.03	4
$HCN\cdots C_{10}H_7GaF_2$	-12.12	52.25	-37.46	58	-24.09	37	-2.82	4
$HCN\cdots C_{10}H_7InF_2$	-10.85	38.87	-30.79	62	-16.65	33	-2.29	5
$HCN\cdots C_{10}H_7TlF_2$	-5.95	32.04	-23.85	63	-11.72	31	-2.42	6
$HCN\cdots C_{10}H_6NH_2AlF_2$	-10.72	37.84	-27.90	57	-17.54	36	-3.12	6
$HCN\cdots C_{10}H_6NH_2GaF_2$	-5.50	37.48	-25.33	59	-14.62	34	-3.02	7
$HCN\cdots C_{10}H_6NH_2InF_2$	-7.20	30.92	-23.55	62	-12.11	32	-2.46	6
$HCN\cdots C_{10}H_6NH_2TlF_2$	-7.26	23.91	-18.73	60	-9.06	29	-3.38	11

Values in parentheses express the percent contribution to the sum of all attractive energy terms.

TABLE S3. EDA/BLYP/ZORA/TZ2P decomposition of the total DFT-D interaction energy with NC^- anion into Pauli repulsion (E_{Pauli}), electrostatic (E_{elec}) and orbital (E_{oi}) interactions, and dispersion correction (E_{disp}). All in kcal mol^{-1}.

	ΔE	E_{Pauli}	E_{elec}	%	E_{oi}	%	E_{disp}	%
$NC^-\cdots C_{10}H_7BF_2$	-84.58	191.65	-138.22	50	-134.95	49	-3.06	1
$NC^-\cdots C_{10}H_7AlF_2$	-90.22	88.35	-113.06	63	-63.09	35	-2.41	1
$NC^-\cdots C_{10}H_7GaF_2$	-83.32	129.08	-132.72	62	-77.27	36	-2.40	1
$NC^-\cdots C_{10}H_7InF_2$	-80.52	114.19	-128.47	66	-64.25	33	-1.99	1
$NC^-\cdots C_{10}H_7TlF_2$	-71.64	147.50	-141.72	65	-75.57	34	-1.85	1
$NC^-\cdots C_{10}H_6NH_2BF_2$	-78.93	183.02	-134.06	51	-125.37	48	-2.51	1
$NC^-\cdots C_{10}H_6NH_2AlF_2$	-70.60	82.92	-96.01	63	-54.59	36	-2.92	2

$NC^{-}\cdots C_{10}H_6NH_2GaF_2$	-87.22	128.98	-135.97	63	-77.49	36	-2.74	1
$NC^{-}\cdots C_{10}H_6NH_2InF_2$	-75.48	110.21	-121.63	66	-61.60	33	-2.46	1
$NC^{-}\cdots C_{10}H_6NH_2TlF_2$	-68.72	138.83	-133.30	64	-71.86	35	-2.39	1

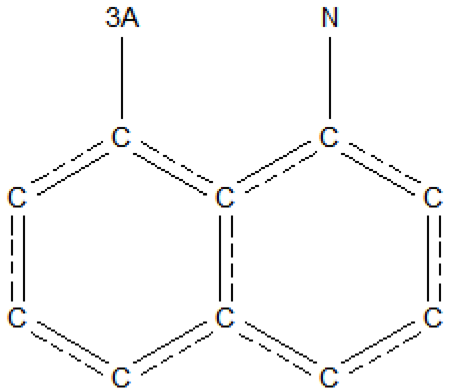

TABLE S4. Number of cases identified in the CSD database for which the internal T(Tr···N) distance lies between the indicated percentage of the sum of covalent radii with an upper limit of the sum of vdW radii.

R_{min}	B	Al	Ga	In	Tl	**Σ**
110%	13	2	2	2	1	**20**
120%	10	0	0	1	1	**12**
130%	9	0	0	0	0	**9**
140%	9	0	0	0	0	**9**

TABLE S5. Examples drawn from the CSD database [taken from ref. 110]. Distances in Å, angles in degs.

CSD refcod	Tr	Tr···N	θ(C2C3N)	θ(C2C1Tr)	Tr···LB	structures

ALAGOJ	B	1.978	112.2	114.4	-	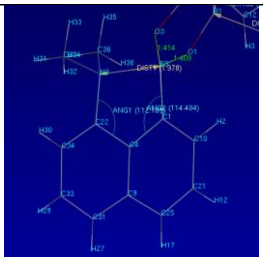
ALAGUP	B	1.832	112.6	112.1	-	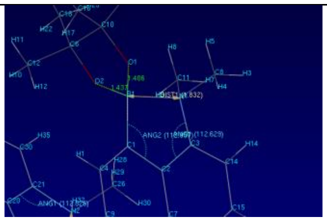
DAFXUF	Ga	2.150	116.9	111.1	-	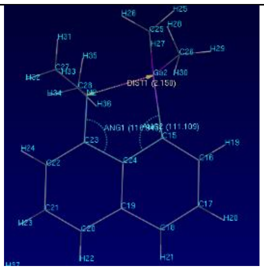
FUPZOH	B	2.866	119.6	128.5	-	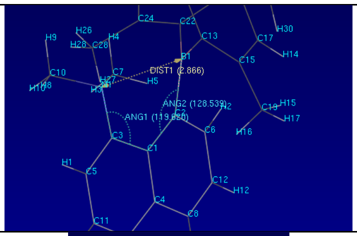
FUPZUN	B	1.726	111.5	109.0		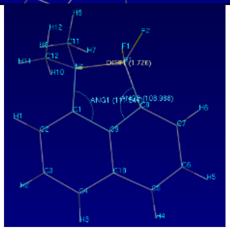
FUQBAW	B	2.773	117.1	125.4	-	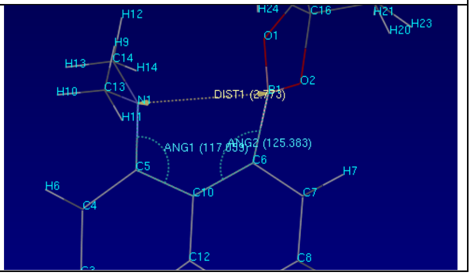

FUQBEA	B	3.198	120.9	130.0	-	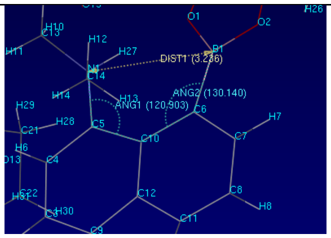
FUQBIE	B	3.025	119.3	128.9	-	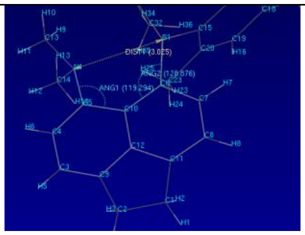
FUQBOK	B	3.170	121.1	129.0	-	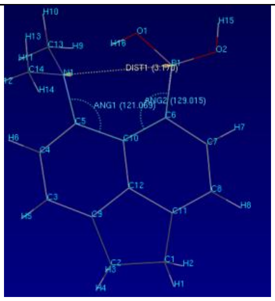
FUQBUQ	B	3.140	119.7	129.6	-	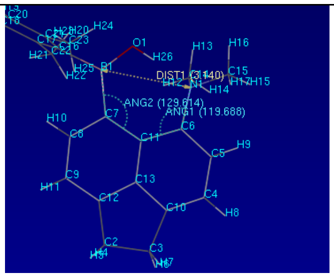
FUQCEB	B	3.077	121.0	126.3	-	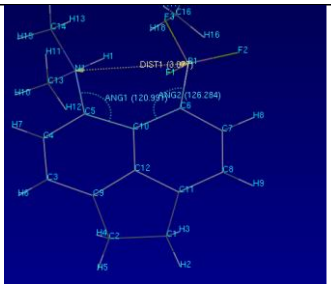

MECNAJ	Al	2.016	116.2	107.5	-	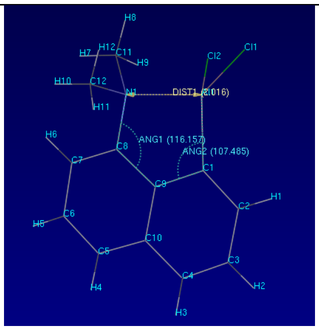
MECNEN	Ga	2.071	115.9	108.0	-	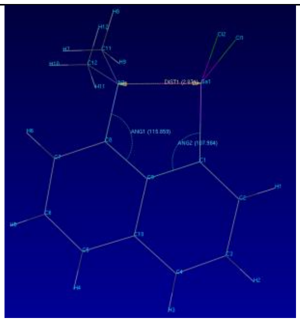
**MECNIR**	**In**	**2.406**	**118.0**	**112.9**	**In···Cl 2.764**	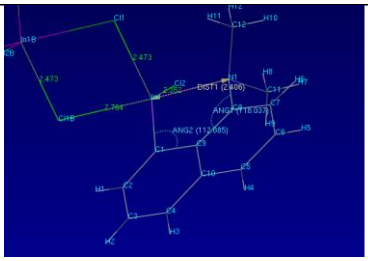
**MECNOX**	**Al**	**2.118**	**115.4**	**110.9**	**Al···Al 2.758**	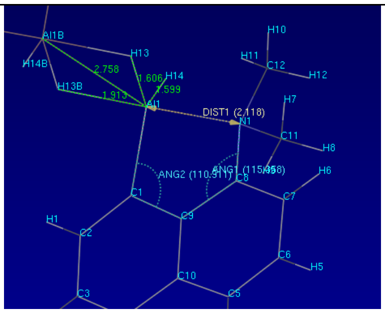
OBODIT	Al	2.068	115.7	109.3	-	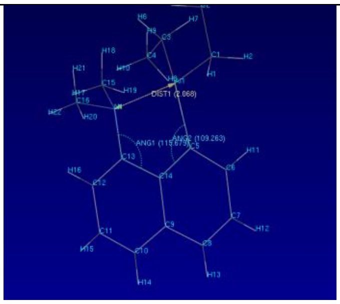

**OBODOZ**	**Al**	**2.265**	**113.8**	**108.6**	**Al···N 2.244**	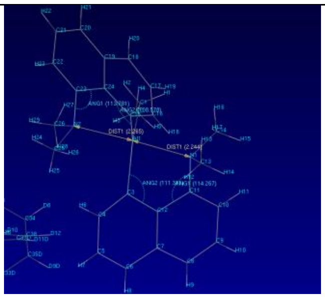
**OGAPIV**	**B**	**1.809**	**118.9**	**132.3**	**B···N 2.941**	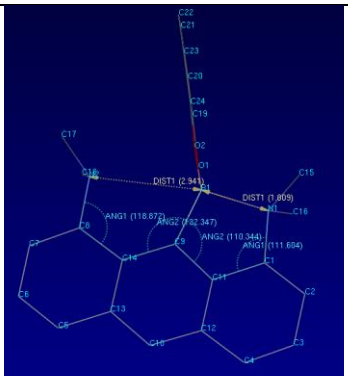
**OGAPOB**	**B**	**1.740**	**118.1**	**135.9**	**B···N 3.123**	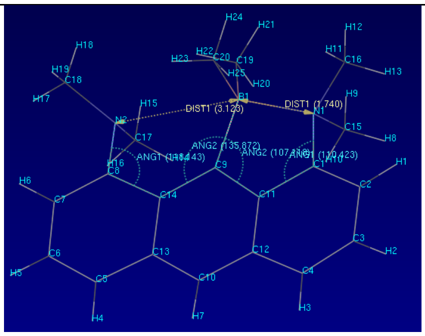
**OGAPUH**	**B**	**1.668**	**118.1**	**136.7**	**B···N 3.128**	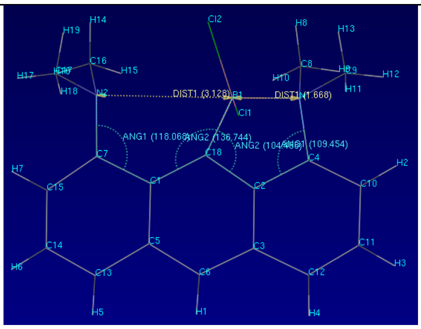

QEHFAM	B	1.742	110.6	109.6	-	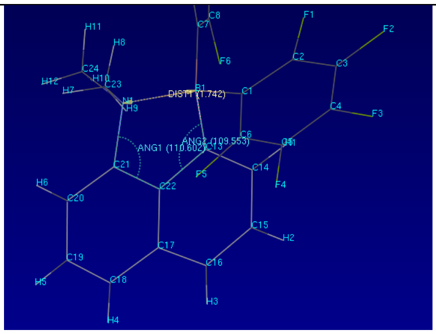
WONZUU	Al	2.056	115.4	108.6	-	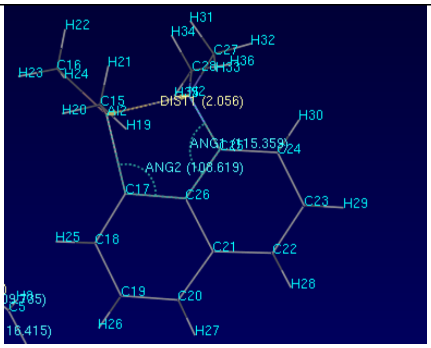
**WUQCUI**	**Tl**	**2.701**	**119.4**	**119.7**	**Tl···N 2.711**	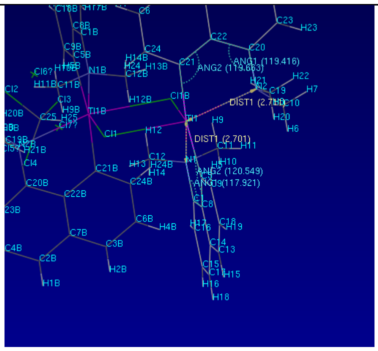
**WUQDAP**	**In**	**2.550**	**189.0**	**117.8**	**In···N 2.620**	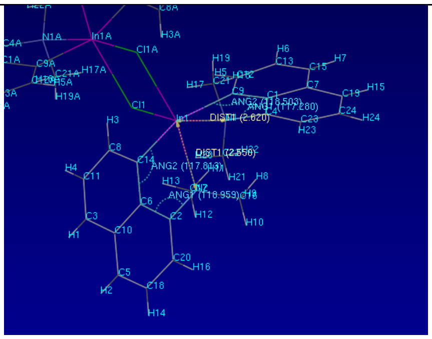
